# Transcriptome Profiling Reveals the Effects of Nitric Oxide on the Growth and Physiological Characteristics of Watermelon under Aluminum Stress

**DOI:** 10.3390/genes12111735

**Published:** 2021-10-29

**Authors:** Yangxia Zheng, Jiachang Xiao, Kaimin Zheng, Junying Ma, Maolin He, Jie Li, Mengyao Li

**Affiliations:** College of Horticulture, Sichuan Agricultural University, Chengdu 611130, China; zhengyx13520@sicau.edu.cn (Y.Z.); scnydxxjc@163.com (J.X.); zhengkaimin111@163.com (K.Z.); ma19993888137@163.com (J.M.); hemaolin821@163.com (M.H.); li1272135958@163.com (J.L.)

**Keywords:** transcriptome, watermelon, nitric oxide, aluminum stress, physiological characteristics

## Abstract

Excessive aluminum ions (Al^3+^) in acidic soil can have a toxic effect on watermelons, restricting plant growth and reducing yield and quality. In this study, we found that exogenous application of nitric oxide (NO) could increase the photochemical efficiency of watermelon leaves under aluminum stress by promoting closure of leaf stomata, reducing malondialdehyde and superoxide anion in leaves, and increasing POD and CAT activity. These findings showed that the exogenous application of NO improved the ability of watermelon to withstand aluminum stress. To further reveal the mitigation mechanism of NO on watermelons under aluminum stress, the differences following different types of treatments—normal growth, Al, and Al + NO—were shown using de novo sequencing of transcriptomes. In total, 511 differentially expressed genes (DEGs) were identified between the Al + NO and Al treatment groups. Significantly enriched biological processes included nitrogen metabolism, phenylpropane metabolism, and photosynthesis. We selected 23 genes related to antioxidant enzymes and phenylpropane metabolism for qRT-PCR validation. The results showed that after exogenous application of NO, the expression of genes encoding *POD* and *CAT* increased, consistent with the results of the physiological indicators. The expression patterns of genes involved in phenylpropanoid metabolism were consistent with the transcriptome expression abundance. These results indicate that aluminum stress was involved in the inhibition of the photosynthetic pathway, and NO could activate the antioxidant enzyme defense system and phenylpropane metabolism to protect cells and scavenge reactive oxygen species. This study improves our current understanding by comprehensively analyzing the molecular mechanisms underlying NO-induced aluminum stress alleviation in watermelons.

## 1. Introduction

Watermelon (*Citrullus lanatus* (Thunb.) Matsum. et Nakai) is an annual vine belonging to the Cucurbitaceae family and widely cultivated all over the world [1]. According to the FAO data in 2019 (https://scienceagri.com/10-worlds-biggest-watermelon-producing-countries/, accessed on 27 October 2021), China is the world’s largest producer of watermelon, with the planting area, output, and consumption ranking first in the world. In recent years, it has been reported that the acidification of the soil led to increased mobile aluminum contents, being one of the reasons for continuous cropping obstacle of watermelon [2]. Importantly, aluminum stress has been documented to inhibit the growth and development of watermelon on acid soil and seriously affect the yield and quality.

Acidic soil accounts for about 30% of the world’s ice-free land area [3]. Given that acidic soils are widely distributed in southern China, aluminum toxicity in acidic soils has been reported as a significant factor limiting local crop production [4]. The most notable feature of aluminum toxicity is that it inhibits the growth of plant root tips and causes damage to root tip cells, thus affecting the absorption of mineral elements and finally stunting plant growth [5]. To adapt to aluminum toxicity in acidic soils, plants have evolved various resistance mechanisms, mainly external rejection and internal aluminum tolerance mechanisms [6]. External aluminum rejection mechanisms predominantly involve the chelation of malic acid, citric acid, and oxalate with Al^3+^ to form non-toxic compounds in the rhizosphere, preventing aluminum ions from entering root tip cells. In contrast, the aluminum tolerance mechanism involves detoxifying and isolating aluminum from vacuoles and transferring aluminum ions that enter the cell to the vacuole to reduce aluminum toxicity.

Nitric oxide (NO) is a small signal molecule that exists as free radicals in plants. It is considered a new type of gas transfer element that can regulate plant growth and development and transmit information to improve the resilience of plants under stress [7,8]. Importantly, NO has been reported to be directly involved in regulating plant growth and stress response. In this regard, studies have shown that any stress could stimulate the production of reactive oxygen species (ROS) in plants, and NO could react with various forms of oxygen to reduce ROS accumulation in plants [9]. NO could also improve the activity of antioxidant enzymes in plants, remove ROS produced in vivo, and ultimately reduce malondialdehyde (MDA, a membrane peroxidation product) levels. In addition, NO has also been documented to delay degradation of chlorophyll content, enhance photosynthesis, regulate the stomatal aperture, and reduce damage to the photosynthetic electron transport chain under stress conditions [10,11]. Current studies have shown that NO could promote root elongation and growth of soybean, rice, and Arabidopsis under aluminum stress and reduce the accumulation of aluminum ions in root tips [12,13,14]. However, the molecular mechanisms underlying NO-induced aluminum stress in watermelon alleviation remain unclear. In the present study, plant physiology and molecular biology techniques were combined to explore mechanisms underlying the effects of exogenous NO on watermelon seedlings under aluminum stress conditions. These findings will help improve our understanding by comprehensively analyzing the mechanisms underlying NO-induced aluminum stress alleviation in watermelons.

## 2. Materials and Methods

### 2.1. Plant Material and Experimental Design

Evenly sized, healthy and plump watermelon seeds were selected (variety Zaojia 8424, produced by Shandong Shouhe seed industry; hybrid; characteristics: medium plant growth, strong disease resistance, planted in a large area in south China), soaked in water at 55 °C for 15 min, cooled to room temperature for another 6 h, and then placed in an incubator at 30 °C to accelerate germination. After germination, we sowed the seeds in a hole dish (the volume ratio of perlite, vermiculite, and organic fertilizer was 4:3:1), and transplanted the seedlings to a 10 × 10 cm nutrition bowl when they grew one leaf. The roots of watermelon seedlings with three leaves were treated with 50 mL treatment solution of different concentrations (CK: normal growth; Al: 1200 μmol/L Al_2_(SO_4_)_3_·18H_2_O, pH = 4.5; Al + NO: 1200 µmol/L Al_2_(SO_4_)_3_·18H_2_O +100 µmol/L SNP, pH = 4.5). After three days of treatment, samples were taken and stored at −80 °C after treatment with liquid nitrogen for later use. Each treatment was set up for three biological replicates. The test site was the Plant Factory at the College of Horticulture, Sichuan Agricultural University. The temperature, light intensity, relative humidity, and light/dark period were set as 25 °C, 5000 lx, 75%, and 16 h/8 h, respectively.

### 2.2. Determination of Growth Indicators

After ten days of treatment, five seedlings were randomly selected for growth index determination for each treatment. The root length and plant height were measured with a ruler (the dividing point was the node position of the rhizome), and the fresh weight and dry weight of the seedlings were weighed and recorded with an electronic scale.

### 2.3. Determination of Leaf Index

#### 2.3.1. Determination of Chlorophyll Fluorescence and Photosynthetic Parameters

After three days of treatment, chlorophyll fluorescence and photosynthetic parameters were measured. The chlorophyll fluorescence parameters of the second true leaf of watermelon seedlings were measured using a chlorophyll fluorescence analyzer (PAM2500, Walz, Nuremberg, Germany). The leaves of watermelon seedlings were placed in a dark room for 30 min after dark adaptation to determine the slow fluorescence induction curve. 

The net photosynthetic rate (Pn), stomatal conductance (Gs), intercellular CO_2_ concentration (Ci), and transpiration rate (Tr) of the third fully expanded leaf (from top to bottom) were determined using an infrared gas analyzer portable photosynthesis system (Li-6400; LI-COR, Inc., Lincoln, NE, USA) between 10 a.m. and 11 a.m. The cuvette conditions for data measurement consisted of photosynthetic photon flux density (PPFD) of 1000 μmol photons m^−2^s^−1^, relative humidity at 60–70%, temperature of 25 °C, and external CO_2_ concentration of 400 ± 10 μmol mol^−1^.

#### 2.3.2. Determination of Stomata and Leaf Tissue Sections

After three days of treatment, the second watermelon leaf was completely unfolded, and colorless nail polish was applied to the middle part of the lower epidermis of leaves by the imprinting method. After 20 min, the nail polish flakes were completely dried with tweezers and made into temporary sections [15]. Stomatal characteristics were observed by optical microscope (Nikon Eclipse E100, Nikon, Tokyo, Japan). Stomatal density, the length (a) and width (b) of stomatal apparatus, stomatal aperture (M), and stomatal area (S) were measured using ImageJ software. The stomatal aperture and stomatal area were calculated using the following formula:

M = b/a, S = Π × a × b/4,

where a is the length, b is width, and Π is 3.14.

The middle part of the watermelon leaf was selected (the midrib avoided) and cut into rectangular pieces of about 1 cm×1 cm. After fixation with FAA (formaldehyde-acetic acid alcohol), a series of operations, including dehydration, wax immersion, and embedding, was performed, and a paraffin section was made. A microtome (RM2016, leica, Frankfurt, Germany) was used to cut 6 µm-thick slices. After spreading, baking, dewaxing, and Safranin O/fast green staining of the wax slices, the characteristics of the cross-section of the leaf were observed under an optical microscope, using an imaging system (Nikon DS-U3, Nikon, Tokyo, Japan). Image acquisition and analysis were performed, and the thickness of the leaf, epidermis, and mesophyll were measured using ImageJ microscopy software.

### 2.4. Determination of Physiological Indicators

After five days of treatment, fully unfolded mature leaves were selected for measurement. The measured indicators included chlorophyll content, superoxide dismutase activity, peroxidase activity, catalase activity, malondialdehyde content, electrolyte permeability, superoxide anion [16], nitrate reductase activity [17], and nitrogen element content [18]. SPSS22.0 was used for significance analysis, and Orign2019b was used for mapping.

### 2.5. RNA Extraction, Library Construction, and Sequencing

After the watermelon leaf sample was ground into powder with liquid nitrogen, Trizol extract was used to extract total RNA (Invitrogen, Carlsbad, CA, USA), according to the manufacturer’s instructions. A spectrophotometer was used to detect the quality and purity of the RNA, and 1.5% agarose gel electrophoresis was used to detect the integrity of the RNA (Table S1). Oligo (dT) magnetic beads were used to enrich the mRNA with polyA. Randomly interrupted mRNA fragments were then used as a template to synthesize the first cDNA strand. Finally, a buffer solution, dNTPs, RNaseH, and DNA polymerase I were added to synthesize the second cDNA. The purified double-strand was enriched by PCR to obtain a cDNA library. Finally, Beijing Novogene Bioinformatics Technology Co. Ltd. (Beijing, China) was commissioned to perform high-throughput sequencing based on the Illumina Hiseq 2500 technology sequencing platform.

### 2.6. Sequence Assembly and Gene Annotation

Data filtering was performed on the raw reads produced by the Illumina Hiseq 2500 sequencing platform to obtain high-quality clean reads by removing linker sequences and low-quality reads. The draft genome sequence of watermelon was used as a reference (http://cucurbitgenomics.org/ftp/genome/watermelon/97103/v2/, accessed on 5 March 2021). Trinity software was used to merge and assemble all clean reads to obtain the genes. DEG sequences were compared to GO and KEGG databases to obtain annotation results.

### 2.7. Screening and Classification of DEGs

EBSeq was used for differential expression analysis to obtain DEG data sets from the two libraries. The False Discovery Rate (FDR) was used as a key indicator for the screening of DEGs. During the screening process, FDR (q-value) < 0.05 and |log2(foldchange)| ≥ 0 were used as the screening criteria.

### 2.8. Verifying Gene Expression Levels by qRT-PCR

The expression levels of 23 genes were verified by qRT-PCR, and primer Premer6 was used to design specific primers (Table S2). The cDNA of CK, Al, and Al + NO were used as the template. The reaction procedure consisted of pre-denaturation at 95 °C for 30 s, then denaturation at 95 °C for 5 s and annealing at 55 °C for 1 min. Dissolution curves were drawn after 40 cycles to determine the specificity of the primers. The experiment was repeated three times. The relative expression levels of target genes were normalized by the geometric mean of ClYLS8 expression levels and then calculated using the 2^−ΔΔCT^ method [19].

## 3. Results

### 3.1. Effects of NO on Watermelon Growth under Aluminum Stress

**Figure 1:**
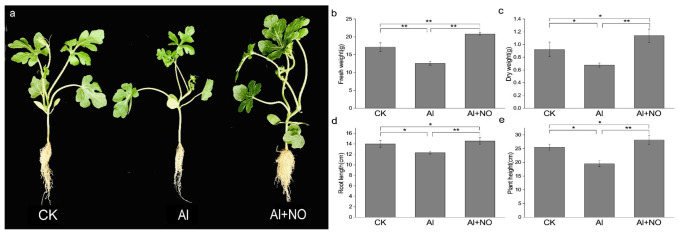
Effects of NO on watermelon growth under aluminum stress. (**a**) Phenotypes of plants following different treatments. (**b**–**e**) Measurements after three days for (**b**) fresh weight, (**c**) dry weight, (**d**) root length, and (**e**) plant height. Data are presented as means ± SD of three biological replicates. Asterisks indicate significant differences (* *p* < 0.05, ** *p* < 0.01) between the means of the treatment groups and the control groups.

As illustrated in Figure 1, aluminum stress significantly inhibited the growth and biomass accumulation of watermelon, documented in terms of decreased fresh weight (b), dry weight (c), root length (d), and plant height (e). In contrast, the exogenous application of NO alleviated the adverse effects of aluminum on watermelon growth and significantly increased the plant height, root length, and biomass of watermelon seedlings.

### 3.2. Effects of NO on Photosynthesis of Watermelon under Aluminum Stress

#### 3.2.1. The Effect of NO on Photosynthetic Parameters and Chlorophyll Fluorescence Parameters under Aluminum Stress

**Figure 2:**
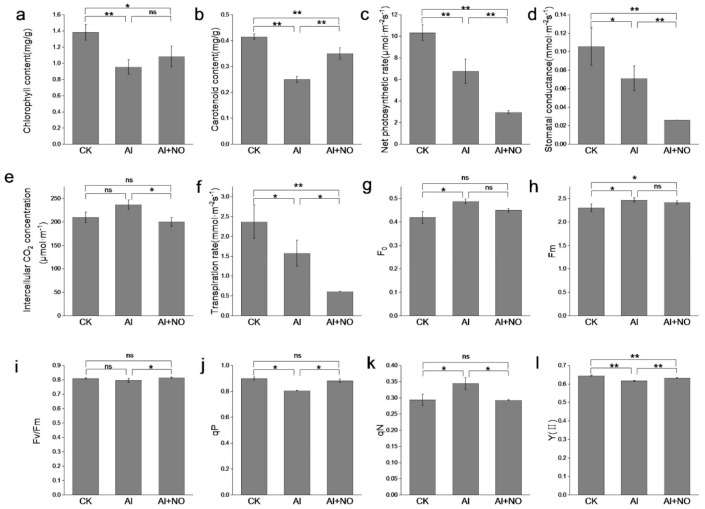
Effects of nitric oxide on photosynthetic parameters and chlorophyll fluorescence parameters of Al-exposed watermelon. (**a**) Chlorophyll content, (**b**) carotenoid content, (**c**) net photosynthetic rate, (**d**) stomatal conductance, (**e**) intercellular CO_2_ concentration, (**f**) transpiration rate, (**g**) F_0_, (**h**) Fm, (**i**) Fv/Fm, (**j**) qP, (**k**) qN, (**l**) Y(II). Data are presented as means ± SD of three biological replicates. Asterisks indicate significant differences (* *p* < 0.05, ** *p* < 0.01, ns *p* > 0.05) between the means of the treatment groups and the control groups.

As seen in Figure 2, the chlorophyll content, carotenoids, net photosynthetic rate, stomatal conductance, and transpiration rate of aluminum-stressed watermelon leaves were significantly reduced compared with CK. The intercellular CO_2_ concentration increased, but there was no statistically significant difference. After the exogenous application of NO, the chlorophyll and carotenoids contents in watermelon leaves were significantly higher than that of Al-exposed watermelon leaves. However, the net photosynthetic rate, stomatal conductance, intercellular CO_2_ concentration, and transpiration rate were significantly lower than in Al-exposed watermelon leaves. This finding indicates that after exogenous application of NO, the stomata of watermelon leaves were closed, and the photosynthetic rate was decreased.

Under aluminum stress conditions, the initial fluorescence (F_0_) and maximum fluorescence (Fm) of watermelon leaves increased significantly compared with CK, and the potential photosynthetic efficiency (Fv/Fm) and actual photosynthetic efficiency (YII) of PSII were decreased. After exogenous application of NO, no significant change in F_0_ and Fm was observed, while Fv/Fm and YII were significantly increased. Leaf photochemical quenching (qP) was 9.42% higher than Al-exposed watermelon leaves, and Non-photochemical quenching (qN) was 15% lower than Al-exposed watermelon leaves. This finding showed that NO could reduce the proportion of photosystem II (PSII) used to dissipate heat, increasing the maximum photosynthetic rate of watermelon seedlings.

#### 3.2.2. Effects of NO on Watermelon Leaf Stomata and Leaf Morphology under Aluminum Stress

**Figure 3:**
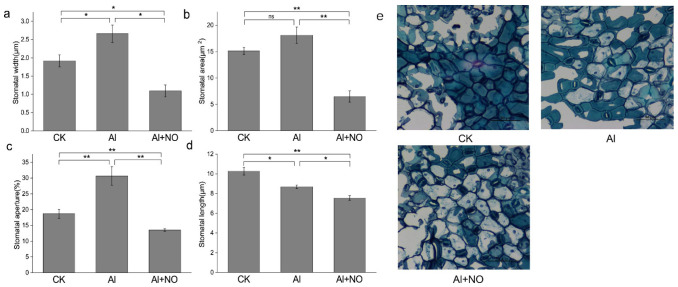
Effects of NO on the stomatal structure of Al-exposed watermelon leaves. (**a**) Stomata width, (**b**) stomata area, (**c**) stomata aperture, (**d**) stomata length, (**e**) the stomatal structure of watermelon leaves following the three different treatments. Data are presented as means ± SD of three biological replicates. Asterisks indicate significant differences (* *p* < 0.05, ** *p* < 0.01, ns *p* > 0.05) between the means of the treatment groups and the control groups.

As shown in Figure 3, after three days of treatment, the largest stomatal width, stomatal area, and stomatal aperture were observed under aluminum stress. After exogenous application of NO, the stomatal length, stomatal width, stomatal area, and stomatal aperture were significantly reduced compared with Al-exposed watermelon leaves. It was found that 80%, 70%, and 30% of the epidermal stomata in CK, Al, and Al + NO were opened in leaf sections, respectively. These results indicate that NO could promote stomatal closure and reduce transpiration dissipation of watermelon leaves under aluminum stress conditions.

**Figure 4:**
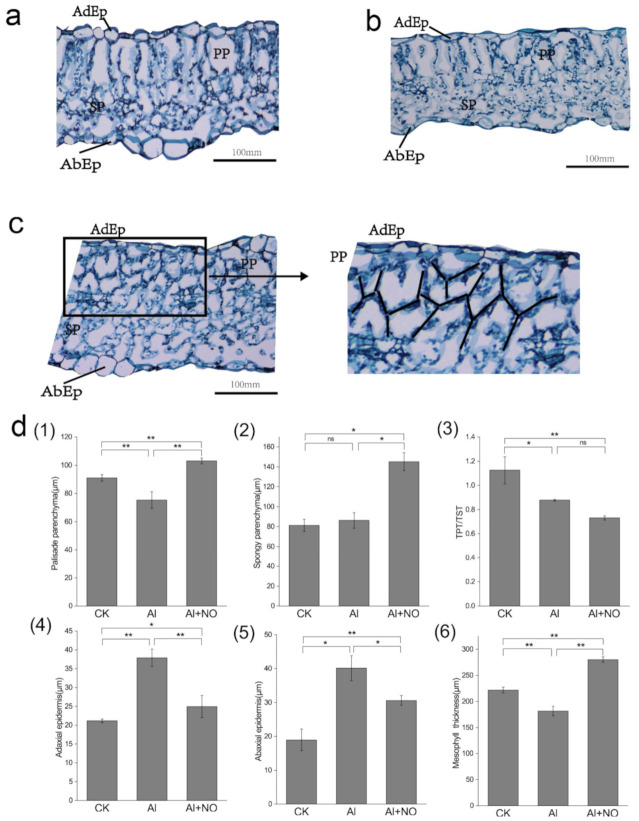
The effect of NO on the morphological structure of watermelon leaves under aluminum stress. (**a**–**c**) Leaf slices of CK, Al, and Al + NO, respectively. (**d**) (**1**) palisade tissue, (**2**) spongy tissue, (**3**) TPT/TST, (**4**) adaxial epidermis, (**5**) abaxial epidermis, (**6**) mesophyll thickness of watermelon leaves under three treatments. Data are presented as means ± SD of three biological replicates. Asterisks indicate significant differences (* *p* < 0.05, ** *p* < 0.01, ns *p* > 0.05) between the means of the treatment groups and the control groups.

As seen in Figure 4, aluminum stress caused changes in the morphology and anatomy of watermelon leaves. Compared with CK, aluminum stress caused a significant decrease in mesophyll thickness, disordered arrangement of palisade tissue, and dense arrangement of sponge tissues in watermelon leaves. After the exogenous application of NO, the thickness of the palisade tissue increased significantly compared with that of Al-exposed watermelon leaves, with a predominant “Y” shape arrangement, while the sponge structure was loosely arranged and significantly thickened. Aluminum stress promoted increased Adaxial and Abaxial epidermis thickness, while the thickness of palisade tissue was significantly reduced by 17.27% compared with CK and the TPT/TST ratio (the ratio of palisade tissue to sponge tissue) was significantly reduced. After the exogenous application of NO, the adaxial and abaxial epidermis thickness decreased significantly, while the mesophyll thickness increased significantly compared with that of Al-exposed watermelon leaves and the palisade tissue and sponge tissue increased by 36.87% and 68.45%, respectively. These findings show that aluminum stress could cause thinning of watermelon leaves, and the exogenous application of NO could promote significant thickening of the leaf mesophyll.

#### 3.2.3. Effects of NO on Oxidation Stress and Nitrogen Metabolism of Watermelon Leaves under Aluminum Stress

**Figure 5:**
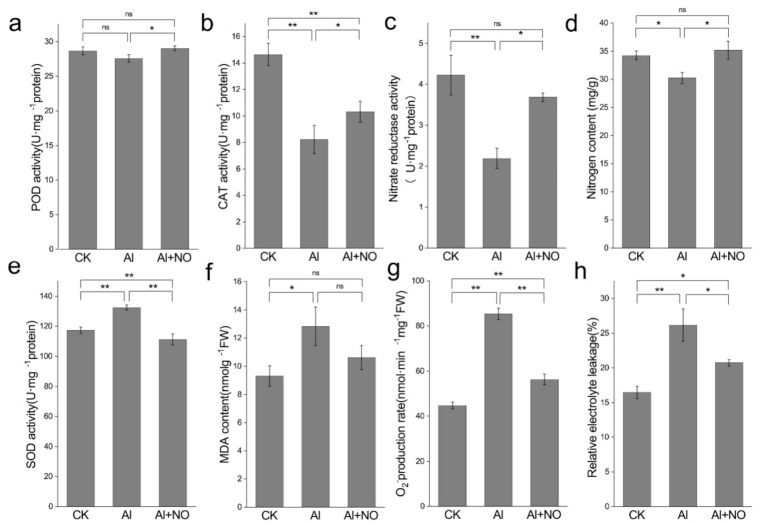
Effects of NO on oxidation stress and nitrogen metabolism of watermelon leaves under aluminum stress. (**a**) POD, (**b**) CAT, (**c**) NR, (**d**) nitrogen content, (**e**) SOD, (**f**) MDA, (**g**) superoxide anion, (**h**) relative electrical conductivity. Data are presented as means ± SD of three biological replicates. Asterisks indicate significant differences (* *p* < 0.05, ** *p* < 0.01, ns *p* > 0.05) between the means of the treatment groups and the control groups.

As seen in Figure 5, compared with CK, aluminum stress inhibited peroxidase (POD), catalase (CAT), and nitrate reductase (NR) activities with decreased accumulation of nitrogen content in watermelon leaves. In contrast, aluminum stress enhanced levels of superoxide dismutase (SOD), malondialdehyde (MDA), superoxide anion content (O2^−^), and increased electrolyte permeability. Exogenous application of NO significantly increased POD and CAT activities, alleviated cell damage under aluminum stress, decreased MDA, O2^−^ content and electrolyte permeability, and promoted NR activity and accumulation of nitrogen content.

### 3.3. Transcriptome Sequencing and Assembly

In order to further explore the mechanisms underlying NO-induced aluminum stress alleviation, nine cDNA libraries (three samples CK, Al, and Al + NO with three biological replicates) were constructed for transcriptome sequencing. A total of 42G of raw data were obtained, and the average Q20 phred score value (an error probability of 1%) of the nine samples was greater than 97%, and the average Q30 phred score value (an error probability of 0.1%) was greater than 92% (Table S3). The watermelon genome data downloaded from http://cucurbitgenomics.org/ftp/genome/watermelon/97103/v2/ (accessed on 5 April 2021) were used as the reference genome for comparison, and the similarity rate of each sample with the reference genome reached 97%. This finding indicated that the transcriptome sequencing quality was high and could be further analyzed.

### 3.4. Analysis of DEGs

**Figure 6:**
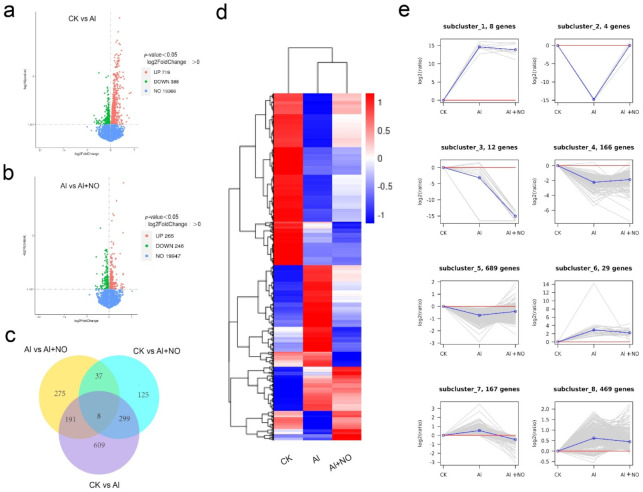
(**a**,**b**) Volcano map of DEGs. (**c**) Venn diagram of DEGs. (**d**) Cluster analysis results of DEGs visualized by a heatmap. The color represents the transcriptional abundance of the DEGs. Red and blue represent high and low expression levels. (**e**) Cluster analysis of DEGs by K-means. The gray line represents the expression of all genes in the cluster.

The transcript abundance of genes was measured in FPKM, and the DEGs were identified based on a false discovery rate (FDR) < 0.05 and |log2 (foldchange)| > 0. The three comparisons (CK vs. Al, Al vs. Al + NO, and CK vs. Al + NO) yielded a total of 1544 DEGs. A total of 1107 (388 upregulated and 719 downregulated) DEGs were identified between CK and Al datasets, while 511 (246 upregulated and 265 downregulated) DEGs were identified between Al and Al + NO datasets (Figure 6a,b).

The intersection between CK vs. Al and CK vs. Al + NO yielded 307 DEGs. After exogenous application of NO, the number of DEGs in CK vs. Al + NO decreased by 57.63% compared with CK vs. Al. This observation suggested that exogenous NO affected watermelon gene expression and reduced the number of DEGs compared with watermelon under aluminum stress.

To further classify the expression patterns of DEGs, the normalized value Z-score after log2 (FPKM) transformation of the 1544 DEGs was used to generate a clustered heatmap (Table S4). As seen in Figure 6e, the DEGs of CK, Al, and Al + NO were further classified into eight clusters based on their expression levels. There were 673 genes upregulated during the comparison between CK and Al datasets and downregulated when Al and Al + NO datasets were compared (subgroups 1, 6, 7, 8). There were 859 genes downregulated when CK and Al datasets were compared and upregulated during Al and Al + NO datasets comparison. According to their upregulated trend, they were divided into three subgroups (subgroups 2, 4, 5). Moreover, 12 differential genes were downregulated during the comparison between CK and Al datasets and Al and Al + NO datasets. These results indicated that the exogenous application of NO resulted in a complex gene expression in response to aluminum stress in watermelon.

### 3.5. Gene Ontology (GO) Classification and KEGG Analysis of DEGs

**Figure 7:**
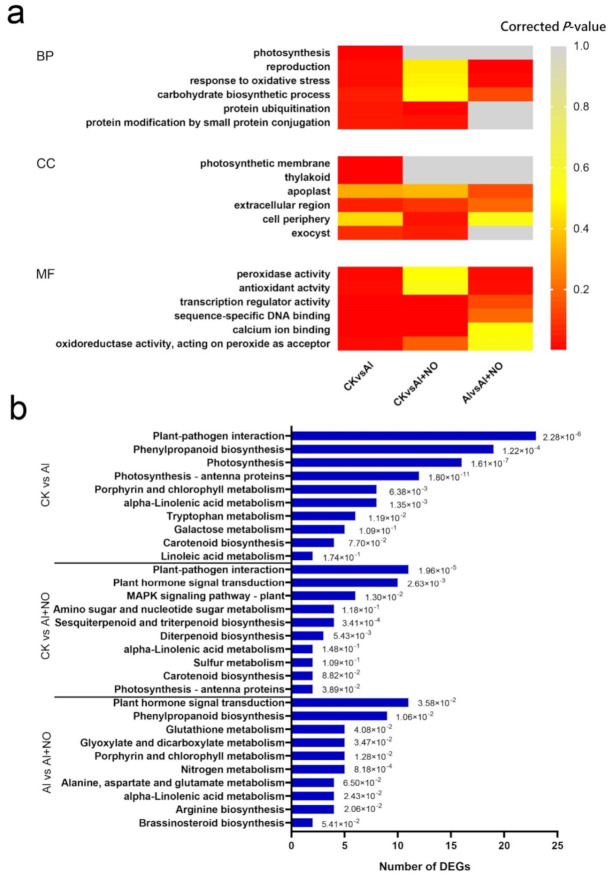
Annotation of DEGs. (**a**) GO enrichment analysis of DEGs. BP, biological process; CC, cellular component; MF, molecular function. Corrected *p*-value < 0.05 denotes significantly enriched terms; the smaller the *p*-value, the closer the color is to red and the more significant the enrichment. (**b**) KEGG enrichment analysis of DEGs. The top 10 enriched terms with highly significant *p*-values (≤0.05) in each comparison are represented.

GO annotation was performed to clarify the main biological functions of DEGs. The CK vs. Al comparison yielded a total of 533 GO terms, of which 294 were associated with biological processes, 39 with cellular components, and 200 with molecular functions (Table S5). Most DEGs were enriched in biological processes (BP), including photosynthesis and response to oxidative stress; cellular components (CC), including photosynthetic membrane and thylakoid; and molecular functions (MF), including peroxidase activity and antioxidant activity, indicating that aluminum stress could affect watermelon photosynthesis, destroy cells’ structure, and cause oxidative damage. The comparison between Al and Al + NO groups yielded 348 GO terms, of which 166 were associated with biological processes, 15 with cellular components, and 167 belonged to molecular functions (Table S6). Among them, most DEGs were enriched in BP such as reproduction and response to oxidative stress, CC such as apoplast and extracellular region, and MF such as peroxidase activity and antioxidant activity, indicating that NO could alleviate the damage caused by aluminum toxicity by regulating the expression of genes involved in oxidative stress, reproduction, and apoplast pathways (Figure 7a). 

We further mapped the DEGs to the Kyoto Encyclopedia of Genes and Genomes (KEGG) database to analyze the metabolic pathways. During KEGG enrichment analysis, we found that 1107 DEGs identified between CK and Al groups were annotated into 89 metabolic pathways, and 511 DEGs obtained from the Al vs. Al + NO comparison were successfully annotated into 65 metabolic pathways. The top 10 pathways associated with enriched differential genes obtained from the two comparisons were statistically analyzed (Figure 7b). The top four enriched pathways during the CK vs. Al comparison were photosynthesis–antenna proteins (5.41%), photosynthesis (7.21%), plant–pathogen interaction (10.36%), and phenylpropanoid biosynthesis (8.55%). The top four enriched pathways obtained from Al vs. Al + NO were all metabolic pathways: nitrogen metabolism (4.50%), phenylpropanoid biosynthesis (8.11%), porphyrin and chlorophyll metabolism (4.50%), and arginine biosynthesis (3.60%). This finding showed that aluminum stress could induce oxidative stress, destroying the chloroplast structure and affecting photosynthesis in watermelon leaves. The exogenous application of NO alleviated aluminum toxicity by influencing plant signal transmission, phenylpropane metabolism, nitrogen metabolism, and other pathways.

### 3.6. Expression Analysis of Antioxidant Enzymes (SOD, POD, CAT) and NR-Related Genes

**Figure 8:**
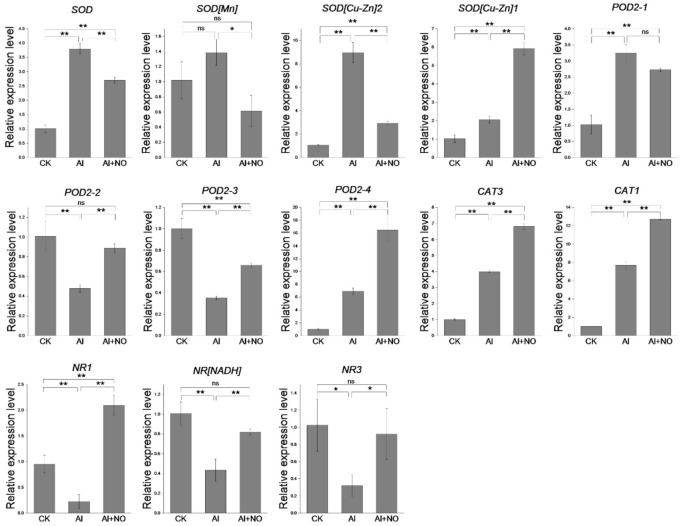
The effect of NO on the expression of three antioxidant enzymes and NR genes in watermelon seedlings under aluminum stress. Data are expressed as the mean ± SD of three biological replicates. Data are presented as means ± SD of three biological replicates. Asterisks indicate significant differences (* *p* < 0.05, ** *p* < 0.01, ns *p* > 0.05) between the means of the treatment groups and the control groups.

It has been reported that aluminum stress could affect the activity of antioxidant enzymes. To further analyze the effect of NO on the expression of related genes, we selected 13 genes for qRT-PCR analysis (Table S7). As seen in Figure 8, *SOD* and *CAT* genes were upregulated, while *POD* genes were down-regulated under aluminum stress conditions. Exogenous application of NO inhibited the expression of genes coding *SOD* and promoted the expression of genes encoding *POD* and *CAT* and three coding *NR* genes, consistent with the results of physiological indexes. The results suggested that NO alleviated aluminum toxicity by increasing antioxidant enzymes and *NR* gene expression levels to relieve oxidative stress and improve nitrogen absorption in watermelon leaves.

### 3.7. Expression Characteristics of Genes Related to Phenylpropanoid Biosynthesis

**Figure 9:**
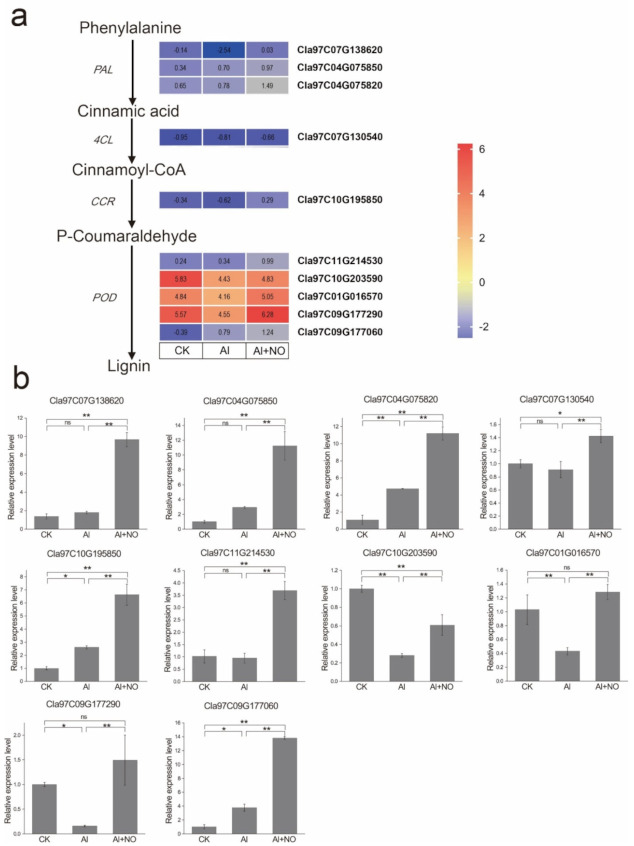
qRT-PCR analysis of DEGs involved in phenylpropanoid biosynthesis following the three types of treatment. (**a**) Heatmap showing the transcriptional abundance of DEGs. FPKM values were log2-based. Red and blue represent high and low expression levels. (**b**) The relative gene expression levels detected by qRT-PCR. The data are presented as the mean ± SD of three biological and technical replicates. Data are presented as means ± SD of three biological replicates. Asterisks indicate significant differences (* *p* < 0.05, ** *p* < 0.01, ns *p* > 0.05) between the means of the treatment groups and the control groups.

Given that phenylpropanoids help plants respond to all aspects of biological and non-biological stimuli, we selected 10 DEGs associated with phenylpropanoid metabolism for qRT-PCR validation. The results showed that Al stress promoted the upregulation of *PAL* and *CCR* genes and inhibited *4CL* gene expression in watermelon leaves. After exogenous application of NO, genes encoding *PAL*, *4CL*, and *CCR* were significantly upregulated compared with Al-treated watermelons. In addition, five DEGs coding for *POD* were significantly upregulated compared with Al-treated watermelons. These results were consistent with the gene expression pattern observed during transcriptome sequencing (Figure 9a,b).

## 4. Discussion

High aluminum soil levels can adversely impact the growth and development of plants, resulting in low quality and yield. In general, low concentration of aluminum can promote the growth of plants, and toxic effects are observed only when the concentration of aluminum exceeds the critical tolerance threshold [20]. The characteristic appearance of aluminum toxicity is inhibition of the elongation and division of root tip cells of plants, resulting in short, coarse, and twisted roots; sparse root hairs and fibrous roots; and enlarged root tips [21]. In the present study, we found that aluminum stress significantly inhibited root elongation and the growth of watermelons, while the addition of NO alleviated growth inhibition, consistent with the literature [22,23].

It is widely acknowledged that photosynthesis plays a significant role in the overall growth of plants, and increased photosynthesis has been reported to promote plant growth [24]. Interestingly, studies have found that NO could alleviate growth inhibition of grapefruit induced by aluminum stress and could reduce damage to the photosynthetic electron transport chain [25]. Meanwhile, NO could increase photosynthesis of cucumber under cadmium stress, improve the light-harvesting complex (LHC) II and LHCI, and increase the chlorophyll fluorescence and pigment content of mustard to maintain plant water content [26,27]. In this study, KEGG annotation showed that the expression of structural genes encoding photosystem II reaction centers (*PsbW*, *PsbQ*, *PsbY*) and photosystem I subunits (*PsaD*, *Psae*, *Psaf*, *Psag*, etc.) was downregulated under aluminum stress, indicating decreased activity of the electron transport chain and weak ability to capture light energy. After exogenous application of NO, the potential photochemical efficiency and the photochemical rate of watermelon leaves were enhanced, and the non-photochemical quenching was reduced, consistent with the previous results. Interestingly, nitric oxide has previously been reported to improve the activity of Rubisco and Rubisco activase in tobacco under copper stress conditions [28] and promote increased ryegrass photosynthetic pigment content, leading to increased photosynthesis and growth [29]. In this study, the chlorophyll content of watermelon increased after exogenous application of NO; however, the net photosynthetic rate decreased. Since a certain linear relationship has been documented between photosynthetic rate and stomatal conductance [30], it was inferred that NO could promote the closure of watermelon leaves, reducing CO_2_ availability and weakening photosynthesis. Furthermore, by observing the cross-section of leaves, it was found that NO could increase the thickness of the watermelon parenchyma and improve the structure of the palisade tissue with a “Y” shape arrangement, which greatly increased the chloroplast surface area that received light and helped to improve the potential photochemical efficiency, further improving watermelon photosynthesis.

Furthermore, high aluminum stress can induce the synthesis of active oxygen free radicals in plants, destroying the balance between free radicals and antioxidant enzymes [31]. If the active oxygen free radicals cannot be scavenged in time, damage to the cell membrane system and plasma membrane peroxidation may occur. Interestingly, a large amount of malondialdehyde reportedly causes electrolyte extravasation to increase conductivity. Studies have shown that NO plays an important role in scavenging superoxide anions and reducing plasma membrane peroxidation. Most importantly, NO can enhance the activity of POD, SOD, and CAT in the root tip of rice under aluminum stress conditions, reduce the accumulation of MDA and O2^·−^, enhance the activity of the antioxidant system of mung bean under cadmium stress, and prevent membrane lipid peroxidation [32,33]. In the present study, the O2^·−^ concentration in watermelon leaves increased significantly under aluminum stress, which caused plasma membrane peroxidation, leading to a significant increase in MDA and electrical conductivity. After the exogenous application of NO, the activity of POD and CAT enzymes was increased while the expression of genes encoding *POD* and *CAT* was significantly upregulated, consistent with the physiological results.

It is widely acknowledged that nitrate reductase (NR) plays a crucial role in catalyzing nitrate reduction and nitrogen metabolism. Accordingly, nitrate reductase activity can be used to quantify nitrogen metabolism [34,35]. During annotation analysis, we found that many DEGs were significantly enriched in the nitrogen metabolism pathway. By analyzing the relative expression levels of three genes that encode *NR*, we found that aluminum stress could inhibit *NR* gene expression, while NO significantly promoted the upregulation of *NR* gene expression. In addition, NR activity and nitrogen content of watermelon leaves under aluminum stress significantly decreased compared with CK, while exogenous application of NO promoted NR activity and nitrogen content. This finding suggests that NO could affect nitrate reductase activity by regulating *NR* gene expression to promote nitrogen absorption.

Phenylpropanoid metabolism plays an important role in plant development and its ability to cope with adversity. The synthesized metabolites mainly include lignins and flavonoids [36]. In this regard, the major function of lignins is to increase the mechanical strength and rigidity of the cell wall and promote the formation of xylem ducts to transport water and nutrients over long distances [37], while flavonoids have been reported to reduce oxidative damage caused by reactive oxygen species [38]. In our study, we found that DEGs were significantly enriched in phenylpropanoid metabolism after exogenous application of NO, and genes encoding *PAL*, *4CL*, and *CCR* were significantly upregulated. Among them, *PAL* and *4CL* are key enzymes involved in plant phenylpropanoid metabolism, which provide precursors for synthesizing downstream metabolites [39]. Furthermore, *CCR* is a key enzyme for lignin synthesis, which converts hydroxycinnamyl-CoA ester into the corresponding hydroxycinnamaldehyde, which plays an important role in coping with biological stress [40,41]. Importantly, class III peroxidases (POD) can reportedly oxidize different substrates with H_2_O_2_ as the electron donor [42]. Studies have shown that the inhibition of class III peroxidases could inhibit the entire lignin biosynthesis pathway [43]. In this study, five genes encoding class III peroxidases were significantly upregulated, indicating that NO could affect the expression of phenylpropanoid metabolism-related genes, promote lignin biosynthesis, and synthesize peroxidases to alleviate oxidative damage induced by aluminum stress to better protect leaf structure and promote watermelon growth. These results indicate that NO plays an important role in alleviating aluminum stress in watermelon, providing new insights into the molecular mechanisms underlying the response in watermelon leaves to aluminum stress, and providing a new approach to tackle the effects of aluminum toxicity in watermelon.

## Data Availability

The transcriptome sequence data generated in this study were deposited at: https://www.ncbi.nlm.nih.gov/bioproject?term=prjna769969.

## Supplementary Materials

The following are available online at https://www.mdpi.com/article/10.3390/genes12111735/s1. Table S1: Concentration of RNA and detection of RIN value. Table S2: Primers used in this experiment and their sequences. Table S3: Transcriptome sequencing quality assessment. Table S4: 1544 DEGs of the three comparison groups. Table S5: GO enrichment analysis of DEGs of CK vs. Al. Table S6: GO enrichment analysis of DEGs of Al vs. Al + NO. Table S7: Transcriptional abundance of three antioxidant enzymes and NR genes.
