# Supplementary material for: Transcriptome Profiling Reveals the Effects of Nitric Oxide on the Growth and Physiological Characteristics of Watermelon under Aluminum Stress
Source: Genes (Basel). 2021 Oct 29;12(11):1735. doi: 10.3390/genes12111735 (PMC8622656; doi:10.3390/genes12111735)
Supplement: Supplementary file 1 [file genes-12-01735-s001.zip › Table S1-3,7.pdf]

Table S1 Concentration of RNA and detection of RIN value.

Sample number	concentration (ng/ul)	volume (ul)	The total (ug)	Integrity value	Detection conclusion
CK-1	539	35	18.865	6.8	A
CK-2	918	35	32.13	6.7	A
CK-3	713	35	24.955	6.8	A
Al-1	834	35	29.19	6.7	A
Al-2	729	35	25.515	6.8	A
Al-3	793	35	27.755	6.9	A
Al+NO-1	692	35	24.22	7	A
Al+NO-2	1117	35	39.095	7	A
Al+NO-3	990	35	34.65	7.1	A

Table S2 Primers used in this experiment and their sequences.

Gene name	Gene ID	Forward Primer(5'-3')	Reverse Primer(5'-3')
SOD	Cla97C10G187890	TCAATCTCTTCCTCTCAGAACAATG	TGCTCAAGTCCACATCAACAC
SOD[Mn]	Cla97C03G050910	TTCAATGGCGGAGGTCACA	ATCAAGGCTTCAAGAGAACCAA
SOD[Cu-Zn]2	Cla97C04G071940	CTTCCATATCCATTCTCTTGGTGAT	CCAGTCGTCTTGCTAAGTTCAT
SOD[Cu-Zn]1	Cla97C02G042100	CGGTGATGATGGTACTGCTACT	TGGTCAGGCTAAGTTCGGTTC
POD2-1	Cla97C01G005010	AACGGCGATTGAACGACTTC	CGAATGCGTCTGGAGTTGTC
POD2-2	Cla97C02G049950	CAAGATGGTATAGTGAGCGAGTT	AAGAGACGATGCCTGGACAA
POD2-3	Cla97C02G049940	ACCGATCTTGTTGCCTTATCTG	ACTTGGTCGCTGGTGAGAA
POD2-4	Cla97C02G049930	CAATGGATGTGATGGCTCTGT	CGCTATTCTACTGTCTCGTCTT
CAT3	Cla97C08G152180	CGCCTTGGACCTAACTATCTTC	TACGAACCGCTCTTGCCTAT
CAT1	Cla97C11G224830	TCGTGTTGGAGGAAGTAATCATAG	CAGGAACTATAATGGCAGGACAA
NR1	Cla97C11G221570	ACTGGAGGTTGAGGTTCTTGA	TTGATTGGCGTCGGTGGAT
NR[NADH]	Cla97C03G060420	GTCGTTCGCTCCTTCAAGTC	TCCTCCTCCTCCTGTTCTATCT
NR[3]	Cla97C03G060430	AGACGACGCAATCCACCTAC	TCCACCTCCACTGACCAGAA
PAL-1	Cla97C07G138620	CAGAGGCACGGATAGTTATGGT	CGTTGTGGTTCAAGAGATTAGCA
PAL-2	Cla97C04G075850	ACTCGTCTGGCTATTGCTTCA	AATATCAATGGCTTCGGCTGTT
PAL-3	Cla97C04G075820	GGTTAGGTCCATTGATTGAAGTGA	CCAGGCGAGTGTTGTCCAT
4CL	Cla97C07G130540	ATTGGCTACATACTGGAGACATTG	AGTGTGCTTCCTGGCTTCTT
CCR	Cla97C10G195850	ATGACTGCTGCCGCTGAAG	TGCTGCTTGTTCTGCCATTGT
POD55	Cla97C11G214530	ATCCGACCACTCCAGACACT	GCTTCCTCATCGCCTCTACAA
POD21	Cla97C10G203590	ACTCCATTGGCATTGATACTGAAG	ATTCTTGATCCACTATGAGCAACC
POD73	Cla97C01G016570	AGTCTCCTGTGCCGATATTCTC	AGTGCGATCATGTCTTGTTGTG
PDON1	Cla97C09G177290	GCTTGTCCTGGCATTGTATCT	GTGAAGTTGAAGAGTCTGTAGTTG
POD53	Cla97C09G177060	AGAACGGCTTGTGGTCATTC	GAGGCCAACACTTCATCCAT
ClYLS8	Cla020175	AGAACGGCTTGTGGTCATTC	GAGGCCAACACTTCATCCAT

Table S3 Transcriptome sequencing quality assessment.

sample	raw_reads	clean_reads	Q20	Q30	GC_pct	total_map
CK_1	44741680	42462940	97.52	92.87	45.34	41668916(97.24%)
CK_2	41150186	39328886	97.65	93.17	45.49	43233770(97.4%)
CK_3	46118962	43694666	97.59	93.06	45.7	41404184(97.49%)
Al_1	45513698	42893180	97.72	93.34	45.54	41841950(97.55%)
Al_2	43501094	41127054	97.55	92.98	45.76	40005418(97.27%)
Al_3	41087334	38884134	97.59	93.03	45.95	37835260(97.3%)
Al+NO_1	45603270	42850482	97.64	93.2	45.92	41315139(97.3%)
Al+NO_2	46908270	44389830	97.52	92.92	45.6	38304143(97.39%)
Al+NO_3	45249970	42472336	97.74	93.35	45.57	42535403(97.35%)

Table S7 Transcriptional abundance of three antioxidant enzymes and NR genes.

Gene name	Gene ID	CK(FPKM)	Al(FPKM)	Al+NO(FPKM)
SOD	Cla97C10G187890	147.5843	202.8647	161.8484
SOD[Mn]	Cla97C03G050910	209.0378	219.7295	193.1981
SOD[Cu-Zn]2	Cla97C04G071940	61.81642	94.56039	83.89868
SOD[Cu-Zn]1	Cla97C02G042100	5.188983	7.134032	6.464014
POD2-1	Cla97C01G005010	0.565454	0.397759	0.835002
POD2-2	Cla97C02G049950	274.8846	93.72426	156.6937
POD2-3	Cla97C02G049940	779.7704	487.9261	665.3568
POD2-4	Cla97C02G049930	14.48687	25.99696	50.24186
CAT3	Cla97C08G152180	3963.484	5162.771	6082.668
CAT1	Cla97C11G224830	313.056	417.6664	506.8128
NR1	Cla97C11G221570	0.525555	0.318489	0.767888
NR[NADH]	Cla97C03G060420	1598.972	1198.411	1692.085
NR[3]	Cla97C03G060430	0.039596	0.060603	0.190951
